# Alteration of leaf shape, improved metal tolerance, and productivity of seed by overexpression of *CsHMA3* in *Camelina sativa*

**DOI:** 10.1186/1754-6834-7-96

**Published:** 2014-06-22

**Authors:** Won Park, Yufeng Feng, Sung-Ju Ahn

**Affiliations:** 1 Bioenergy Research Center, Department of Bioenergy Science and Technology, Chonnam National University, Gwangju, Republic of Korea

**Keywords:** *Camelina sativa*, Bioenergy crop, Heavy metal, CsHMA3, Leaf shape, Productivity

## Abstract

**Background:**

*Camelina sativa* (L.) Crantz, known by such popular names as “gold-of-pleasure” and “false flax,” is an alternative oilseed crop for biofuel production and can be grown in harsh environments. Considerable interest is now being given to the new concept of the development of a fusion plant which can be used as a soil remediation plant for ground contaminated by heavy metals as well as a bioenergy crop. However, knowledge of the transport processes for heavy metals across *Camelina* plant membranes is still rudimentary.

**Results:**

Firstly, to investigate whether *Camelina HMA* (heavy metal P_1B_-ATPase) genes could be used in such a plant, we analyzed the expression patterns of eight *HMA* genes in *Camelina* (taken from the root, leaf, stem, flower, and silique). *CsHMA3* genes were expressed in all organs. In addition, *CsHMA3* was induced in roots and leaves especially after Pb treatment. Heterogeneous expression of *CsHMA3* complemented the Pb- or Zn-sensitive phenotype of *Δycf1* or *Δzrc1* yeast mutant strains. Subsequently, we cloned and overexpressed *CsHMA3* in *Camelina*. The root growth of transgenic lines was better than that in the wild-type plant under heavy metal stress (for Cd, Pb, and Zn). In particular, the transgenic lines showed enhanced Pb tolerance in a wide range of Pb concentrations. Furthermore, the Pb and Zn content in the shoots of the transgenic lines were higher than those in the wild-type plant. These results suggest that overexpression of *CsHMA3* might enhance Pb and Zn tolerance and translocation. Also, the transgenic lines displayed a wider leaf shape compared with the wild-type plant due to an induction of genes related to leaf width growth and showed a greater total seed yield compared to the wild type under heavy metal stress.

**Conclusions:**

Our data obtained from physiological and functional analyses using *CsHMA3* overexpression plants will be useful to develop a multifunctional plant that can improve the productivity of a bioenergy crop and simultaneously be used to purify an area contaminated by various heavy metals.

## Background

Nowadays, the requirement for fossil fuels is increasing exponentially, and it is calculated that the remaining fossil fuel reserves will be consumed by the year 2050 [1-3]. Vegetable oils are widely recognized as promising alternatives to regular diesel fuel, because they are renewable and environmentally friendly. Among the several renewable energy sources, oilseed crops are increasingly being considered as materials to fulfill the rising demand of fossil fuels and their products [4]. *Camelina sativa*, known by its popular names of “gold-of-pleasure” and “false flax,” is an alternative oilseed crop that can be used as a potential low-input coast oil crop for biodiesel production and can be grown under different climatic and soil conditions. Although it was widely grown in Europe and Russia until the 1940s, *Camelina* was largely displaced by higher yielding crops after World War II [5-8].

In recent years, *Camelina* production has increased somewhat due to the heightened interest in vegetable oils that are high in omega-3 fatty acids (a principal component of *Camelina* oil) [9,10]. *Camelina* has a short growing season (85 to 100 d), so it can be incorporated into double cropping systems during the period between harvest and planting times of the main crop. However, the full potential of this crop has not yet been explored, and very little plant breeding or crop production improvement has been performed on *Camelina*.

To meet the increasing demand for oilseed crops, the cultivation of oil crops on a large scale needs to be considered without wasting agricultural land. Therefore, other types of land which we consider as having barren soil can be used for cultivation following a suitable scientific approach.

Heavy metal contamination is a serious problem of increasing significance for environmental reasons. In particular, many crops are exposed to cadmium (Cd) and lead (Pb) around abandoned mines. Therefore, considerable interest has developed recently in the use of terrestrial plants as a green technology for the remediation of surface soils contaminated with toxic heavy metals [11,12]. Interest in phytoremediation has grown significantly following the identification of metal hyperaccumulator plant species. Hyperaccumulators are conventionally defined as plants having a Zn concentration above 10,000 μg g^-1^ dw, a Pb and Co concentration higher than 1000 μg g^-1^ dw, or a Cd concentration above 100 μg g^-1^ dw [13,14].

Based on genetic and functional studies of the *Arabidopsis* P_1B_-ATPases, it is predicted that certain pumps (HMA1 to 4) will be capable of transporting Cd, Pb, Zn, and Co, whereas other pumps (HMA 5 to 8) could transport Cu and potentially Ag [15-19]. Meanwhile, the presence of a multiple genomic copy number can enhance the expression levels of some candidate genes for metal hyperaccumulation and tolerance in hyperaccumulators [20-22]. According to a recent study, genes in *Camelina* were found in two or three copies, and *Camelina* has a hexaploid genome [23]. In this study, we investigated the productivity index between wild-type and *CsHMA3* transgenic lines, and tested whether *Camelina* HMA3 (heavy metal P_1B_-ATPase 3) can enhance functions in metal accumulation or tolerance. The results obtained from the present study provide knowledge on how to improve the growth of a biodiesel species of *Camelina* on different concentrations of heavy metals, such as Cd, Pb, Zn, and Co.

## Results

### Effect of heavy metal stress on physiological responses in *Camelina* and rapeseed

**Figure 1:**
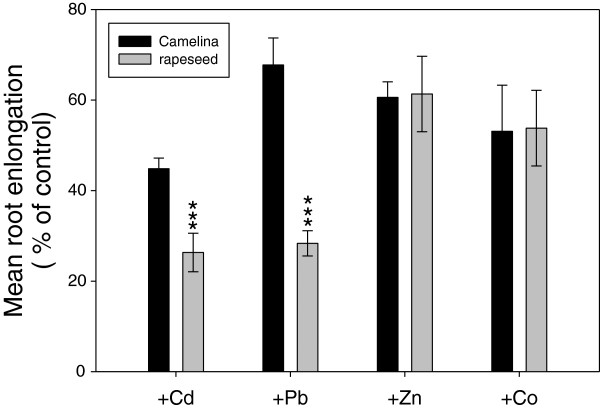
**Effect of metal treatments on root growth in *Camelina *and rapeseed.** Seeds were germinated and grown on agar plates containing 50 μM Cd, 500 μM Pb, 500 μM Zn, and 100 μM Co for seven days. Data represent means ± SD (n = 30). Mean values of root length in the normal medium as control for Cd, Zn, and Co were 4.10 ± 0.69 (*Camelina*) and 4.62 ± 0.53 (rapeseed); mean values of root length in the no-phosphate medium control for Pb were 3.25 ± 0.11 (*Camelina*) and 3.80 ± 0.55 (rapeseed). Asterisks in the figure indicate significant difference between *Camelina* and rapeseed subjected to the same treatment at ***P < 0.001 by Student’s *t-*test.

Rapeseed (*Brassica napus* L.) is another alternative oilseed crop and is known as a potentially useful candidate for phytoremediation [24,25]. To study whether *Camelina* can also be used for phytoremediation, we performed a comparative analysis of the physiological responses of *Camelina* and rapeseed under heavy metal stress. Firstly, we have investigated the effect of metal in a concentration-dependent manner on root growth and germination. Metal-treated root was able to grow with up to 50 μM Cd, 500 μM Pb, 500 μM Zn, and 100 μM Co. Beyond this range, the root growth rate was drastically reduced (data not shown). This result indicates that the critical concentration for growth of both plants is considered to be 50 μM Cd, 500 μM Pb, 500 μM Zn, and 100 μM Co in our system. We also observed that root growth rate and electrolyte leakage were affected by heavy metal treatment in both plants; however, *Camelina* treated with Cd and Pb exhibited less inhibition of root growth rate, and leaves treated with Pb showed a lower amount of leakage than that of rapeseed (Figure 1; Additional file 1: Figure S1).

**Figure 2:**
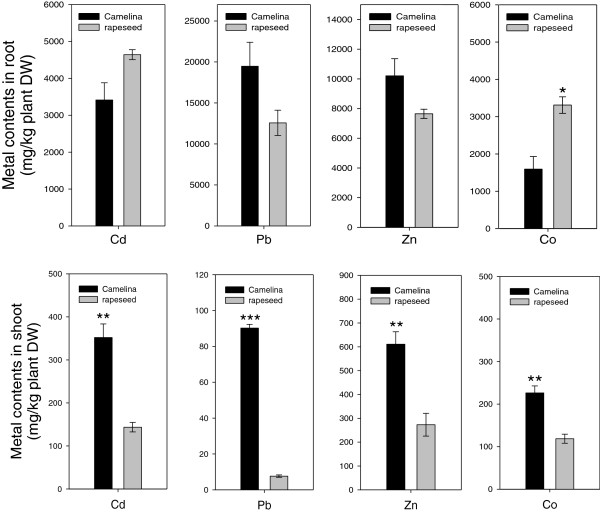
**Metal contents in roots and shoots of *Camelina *and rapeseed*. ***Four-week-old plants were grown hydroponically and treated with 50 μM Cd, 500 μM Pb, 500 μM Zn, and 100 μM Co for one week. Data represent means ± SD (n = 3) from three independent plants. Asterisks in the figure indicate significant difference between *Camelina* and rapeseed subjected to the same treatment at *P < 0.05, **P < 0.01, and ***P < 0.001 by Student’s *t-*test.

To compare the heavy metal uptake ability between *Camelina* and rapeseed, we measured the Cd, Pb, Zn, and Co content in *Camelina* and rapeseed grown in solution with either 50 μM Cd, 500 μM, 500 μM Zn, or 100 μM Co, respectively, for one week (Figure 2). Although the Cd content in roots of rapeseed seems slightly higher than that of *Camelina*, the difference was not statistically significant. Also, no statistical difference was shown in the Pb and Zn contents in roots between *Camelina* and rapeseed. The Co content in *Camelina* root was lower than that of rapeseed. In the shoots, the Cd, Pb, Zn, and Co contents in *Camelina* were higher than those of rapeseed (Figure 2). When comparing physiological parameters for long-term stress between *Camelina* and rapeseed, no statistical differences were observed in chlorophyll content, stomata conductance, and growth rate (data not shown). These results suggest that *Camelina* also has the potential to be used for phytoremediation along with rapeseed.

### Identification and phylogenetic analysis of *CsHMA3* gene

Our next research task involved the possibility of a new transgenic plant that is better suited for heavy metal phytoremediation. To determine if *Camelina* genes could be used in such a plant, the full-length cDNA of *Camelina sativa* L*. HMA3* (*CsHMA3*) was first isolated from cultivar “*CAME*” with 5’ and 3’ PCR primers. Based on the *Camelina* Genome Project Portal (http://www.Camelinagenomics.org and http://www.Camelinagenomics.net; non-public databases), we found that the *CsHMA3* gene occurs in three copies in the genome of *Camelina*, named *CsHMA3a, CsHMA3b,* and *CsHMA3c*. The *CsHMA3* gene sequence obtained in this study was completely consistent with *CsHMA3a*. The cDNA [GenBank: JX402100] was 2286 bp and the *CsHMA3a* encoded polypeptides of 762 amino acids in length and showed 96.5% identity with *CsHMA3b* [GenBank: KJ818901], while the presence of a stop codon at a position of 75 amino acids in length in *CsHMA3c* [GenBank: KJ818902] was observed (Additional file 2: Figure S2).

CsHMA3 belongs to the P_1B_-ATPases and includes the predicted eight transmembrane domains and the key identifying structures of P_1B_-type ATPases found in other subfamily members such as *Arabidopsis* and *Thlaspi* (Additional file 2: Figure S2), suggesting parallel functions in these species [17,22,26].

### Expression pattern of *CsHMAs*

**Figure 3:**
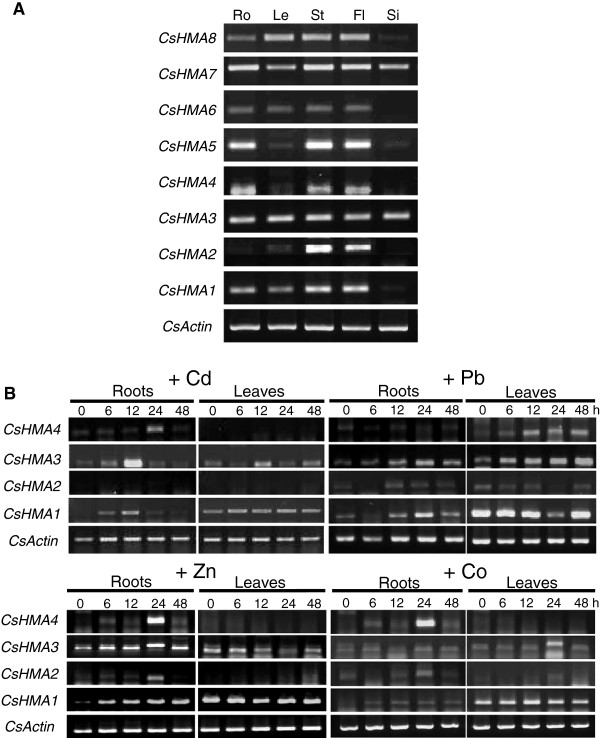
**Expression profile of *CsHMAs.*** RT-PCR analyses were performed using RNAs isolated from various tissues in normal condition **(A)** or from roots and leaves 0, 6, 12, 24, and 48 hours after μM Cd, 350 μM Pb, 350 μM Zn, and 50 μM Co treatment **(B)**. Plants grown hydroponically were used in these experiments. Root (Ro), leaf (Le), stem (St), flower (Fl), silique (Si).

To investigate whether *Camelina* HMAs (heavy metal P_1B_-ATPases) can enhance functions in metal accumulation or tolerance, we first analyzed the expression patterns of eight *HMA* genes in *Camelina* (root, leaf, stem, flower, and silique) under normal conditions by reverse transcription (RT)-PCR. *CsHMA* primers were designed by multiple sequence alignment (Clustal W) in order to amplify a specific cDNA fragment of each gene. The PCR products were sequenced to confirm that only the transcriptions of the consistent gene were amplified. *CsHMA3* and *CsHMA7* genes were expressed in all organs. Expression of *CsHMA1*, *CsHMA6*, and *CsHMA8* genes appeared to be weak only in siliques. *CsHMA4* and *CsHMA5* were mainly detected in the root, stem, and flower. The *CsHMA2* expression pattern was similar to those of *CsHMA4* and *CsHMA5* except for low transcript level in the root (Figure 3A). In *Arabidopsis*, HMA1 to HMA4, which transport Zn^2+^, Cd^2+^, Pb^2+^, and Co^2+^, and AtHMA5 to AtHMA8, which transport Cu^+^ and Ag^+^ were identified by several studies [15-19]. Therefore, we mainly analyzed the expression profiles of *CsHMA1, 2, 3,* and *4* as potential candidate genes for the specific transport or storage of Cd, Pb, Zn, and Co. RT-PCR analyses were performed using RNAs isolated from the roots and leaves of *Camelina* grown in hydroponic culture containing either 50 μM Cd, 500 μM Pb, 500 μM Zn, or 100 μM Co. In some cases up-regulated expression levels of a particular gene could be found. The expression of *CsHMA1* and *CsHMA2* showed elevated levels in Pb-treated roots, and *CsHMA3* seemed to be induced in roots and leaves from 6 hours after Pb treatment. Although the *CsHMA4* genes exhibited unaltered expression levels in roots during Pb treatment, an induction in the leaves was shown from 6 hours after Pb treatment (Figure 3B). Among *CsHMA1* to *CsHMA4*, the expression levels of *CsHMA1* and *CsHMA3* were more highly regulated by Pb, Zn, Co, and Cd (Figure 3B) than others. This data raised the possibility that *CsHMA1* and *CsHMA3* can be used as potential candidates for phytoremediation in future studies. Since HMA3 has been identified in various plants and is an important key factor that enhances metal uptake and effectively detoxifies toxic metals by compartmentation into vacuoles [22,27], we focused on the *CsHMA3* gene in this study.

### Functional characterization of CsHMA3 by heterologous assay in yeast

**Figure 4:**
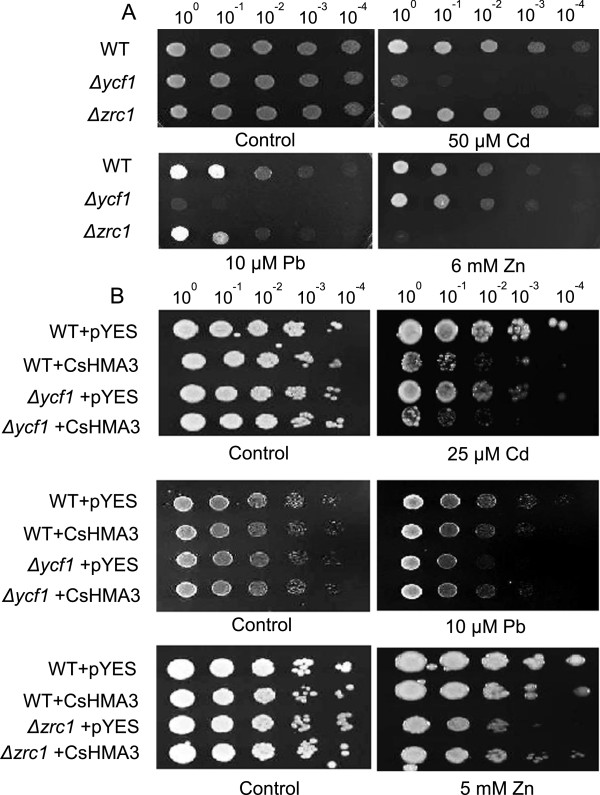
**Functional assay of CsHMA3 of the wild type and mutants in yeast.** Confirmation of Cd and Pb sensitivity of *Δycf1,* and Zn sensitivity of *Δzrc1* mutant strain compared with those of wild-type (BY4741) yeast strain **(A)**. Cell growth of the *Δycf1* or *Δzrc1* mutant strain was transformed with empty vector pYES2, CsHMA3 **(B)**. Yeast cells grown in the log phase were diluted to an OD_600_ of 1, 10^-1^, 10^-2^, 10^-3^, and 10^-4^, and 5 μL of each dilution were spotted onto the 1/2 YPD **(A)** or LPM medium containing galactose **(B)** without or with added CdSO_4_, PbNO_3_, and ZnSO_4_. The plates were incubated at 30°C for four days before photography. The yeast cells were grown in the presence of 25 μM Cd, 10 μM Pb, and 5 mM Zn. The photographs shown are representative results of independent experiments that were performed three times with reproducible results.

To investigate whether CsHMA3 could function in metal tolerance, we performed a yeast functional complementation. The empty vector pYES and the pYES vector containing CsHMA3 were used to transform the yeast *Δycf1* (Cd/Pb-sensitive mutant) or *Δzrc1* (Zn-sensitive mutant). The phenotype of *Δycf1* is caused by the loss of a yeast cadmium factor 1 (YCF1), a vacuolar glutathione S-conjugate of the ATP-binding cassette (ABC) transporter family associated with multidrug resistance in *Saccharomyces cerevisiae*. YCF1 has been reported to contribute to Cd and Pb resistance [28,29]. The zinc resistance conferring1 (ZRC 1) in *S. cerevisiae*, a member of the cation diffusion facilitator (CDF) family that is involved in zinc efflux and compartmentalization, is known to contribute to zinc tolerance. In addition, *Δzrc1* strains are hypersensitive to zinc [30]. Our results confirm that these mutants have a higher metal-sensitive phenotype compared with the wild-type yeast strain (Figure 4A). Although *CsHMA3* expression in the wild-type strain could not enhance the tolerance of Cd, Pb, and Zn, the *Δycf1* or *Δzrc1* mutant strain yeast expressing *CsHMA3* was able to complement the growth defect in the presence of 10 μM Pb and 5 mM Zn, but not in the Cd medium (Figure 4B). Rather the expression of *CsHMA3* in the wild-type strain and *Δycf1* increases sensitivity to Cd. According to a previous report [31], the expression of *AtHMA3* in metal-sensitive yeast strains slightly increases tolerance to Cd and Pb, but not to Zn. The phenotype of *CsHMA3*-expressing strains was partially consistent with the expectation according to AtHMA3 function. Although it is difficult to determine precise metal substrates for CsHMA3 at the current stage, our result suggested that the expression of *CsHMA3* is indirectly responsible for the increased Pb and Zn tolerance in metal-sensitive yeast strains.

### Overexpression of *CsHMA3* in *Camelina* improved Cd, Pb, and Zn tolerance

To test whether CsHMA3 can be used to develop plants with enhanced metal tolerance, we generated transgenic *Camelina* expressing a 35S promoter:: *CsHMA3*, using a gateway cloning system. After confirmation of *CsHMA3* transcript levels of five *CsHMA3a* independent transgenic T_2_ plants (H3-1, 3-2, 3-3, 3-5, and 3-7) and the wild type determined by RT-PCR using a 35S promoter primer and *CsHMA3* specific primer (Additional file 3: Figure S3A), we selected the H3-1 and H3-3 *CsHMA3* transgenic lines, which showed a much higher transcript level as compared to other lines, and then renamed them OX1 and OX2, respectively. In addition, OX3 is H3-2 and OX4 is H3-7. Differences in the phenotypes of wild-type and *CsHMA3*-overexpressing plant leaves were observed. Transgenic lines have more rounded-type leaves, whereas wild-type leaves are sharp (Additional file 3: Figure S3B).

**Figure 5:**
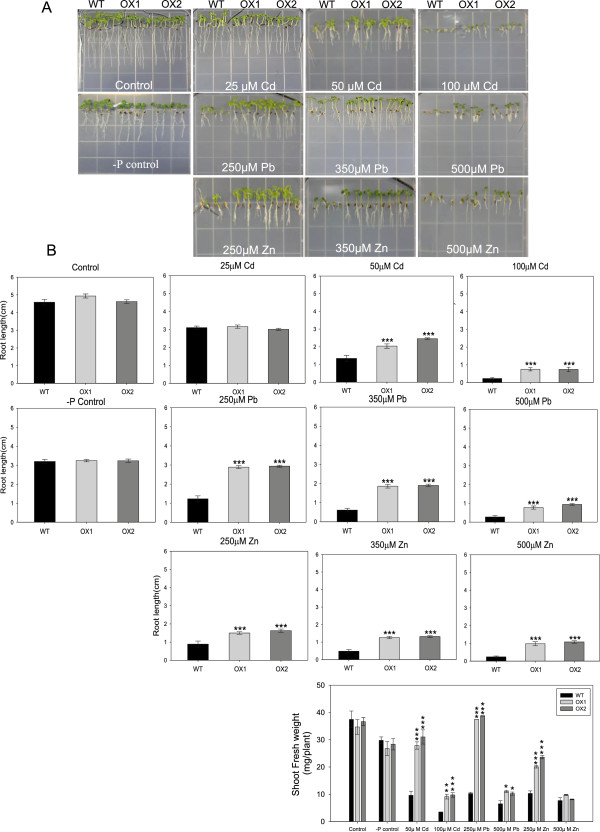
**Effect of metal treatments on root growth and shoot fresh weight in transgenic lines.** Photographs of *Camelina* wild-type and two *CsHMA3*-overexpressing lines grown on agar plates containing different concentrations of Cd, Pb, and Zn stress for one week **(A)**. Root growth rate **(B)** and fresh weight **(C)** of the plants were measured at the seventh day after heavy metal treatment. For the Pb treatment, we used control medium without phosphate. Data represent means ± SD (n = 30). Asterisks in the figure indicate significant difference from wild type at *P < 0.05, **P < 0.01, and ***P < 0.001 by Dunnett’s test.

**Figure 6:**
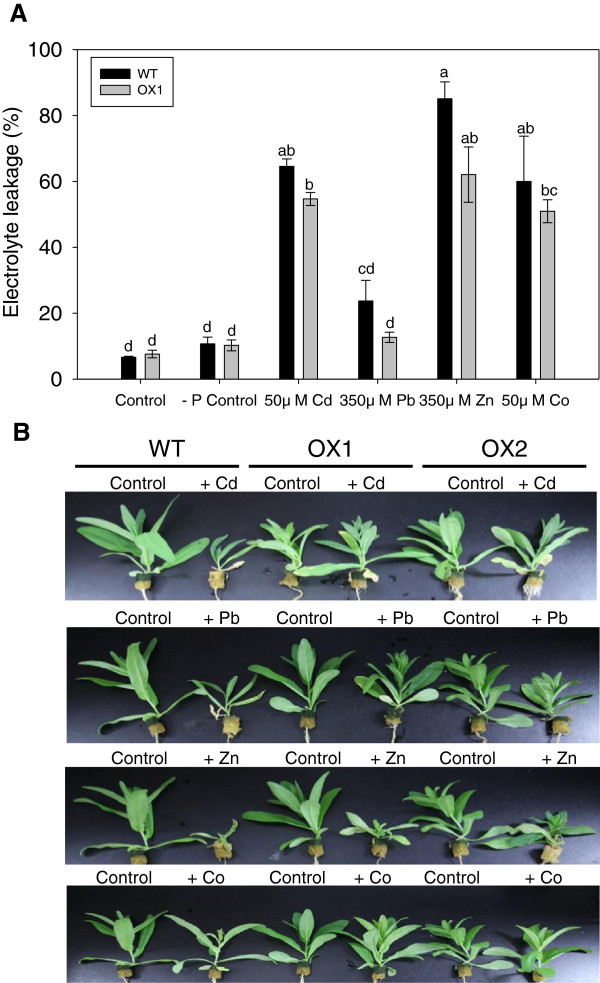
**Effect of metal treatments on the electrolyte leakage of leaves in transgenic lines.** Electrolyte leakage was expressed in percentage (%) of total electrolyte leakage **(A)**. Data represent means with ± SD (n = 3). Different lowercase letters in the figure indicate significant difference at *P* < 0.05 by Tukey’s test. Two-week-old plants were hydroponically grown and treated with 50 μM Cd, 350 μM Pb, 350 μM Zn, and 50 μM Co for seven days **(B)**. For Pb treatment, plants were transferred to phosphate-free nutrient solution at least 12 hours prior to treatment.

To evaluate heavy metal tolerance of *CsHMA3* transgenic lines, seeds of wild-type and two transgenic lines were germinated and grown on agar plates containing 25, 50, or 100 μM Cd, 250, 350, or 500 μM Pb or Zn, and 50 or 100 μM Co for one week. In the case of Pb treatment, we used modified medium without phosphate for control and Pb treatment to avoid Pb precipitation. No differences occurred in the germinations between the wild-type and transgenic lines in treatments compared to the control. The root growth and shoot fresh weight were measured one week after treatment. The transgenic lines grew better than the wild-type lines under Cd, Pb, and Zn stress (Figure 5). In particular, the transgenic lines showed enhanced Pb tolerance in a wide range of Pb concentrations (Figure 5). However, in the control medium and Co-treated medium, the growth of each plant was similar (Additional file 4: Figure S4). In addition, the contribution of overexpression of *CsHMA3* to the enhanced Pb tolerance of *Camelina* was further evaluated by measuring the electrolyte leakage from the leaves of the plants (Figure 6). The leaves from the *CsHMA3* transgenic line showed a lower rate of electrolyte leakage than that observed in the leaves from the wild types.

### *CsHMA3* overexpressing transgenic *Camelina* have increased Pb and Zn contents

**Figure 7:**
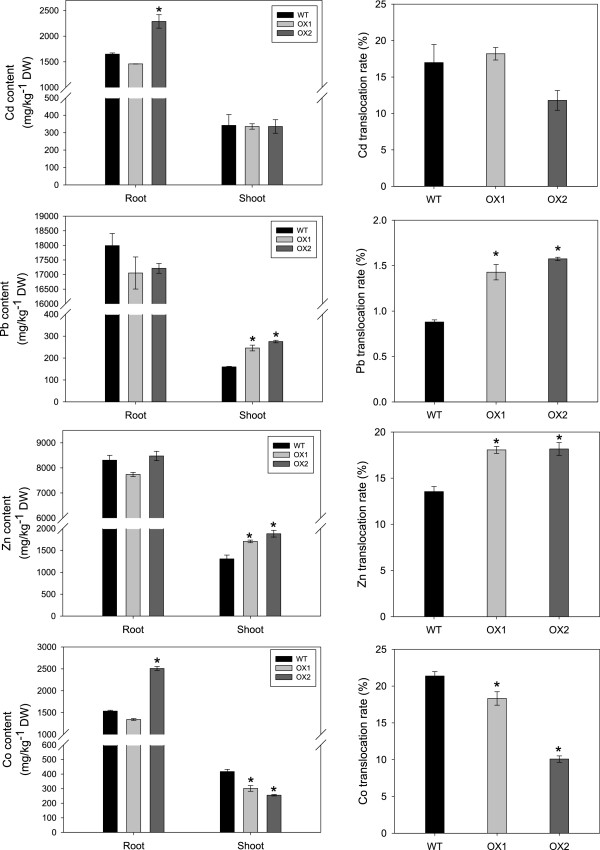
**Metal contents in roots and shoots and the translocation ratio in the transgenic lines.** Three-week-old plants were grown hydroponically and treated with 50 μM Cd, 350 μM Pb, 350 μM Zn, and 50 μM Co for one week. Data represent means ± SD (n = 3). Asterisks in the figure indicate significant difference from wild type at *P < 0.05 by Dunnett’s test.

To analyze the heavy metal uptake ability of the *CsHMA3* transgenic line, we measured the Cd, Pb, Zn, and Co contents in wild-type and overexpressing lines treated with 50 μM Cd, 350 μM Pb, 350 μM Zn, and 50 μM Co in hydroponic culture for one week. Based on the analysis of heavy metal tolerance between the wild-type and *CsHMA3* transgenic lines, we determined appropriate concentrations of heavy metal treatments. The results showed that Pb and Zn contents in shoots of the transgenic line were higher than those of the wild type, but no significant differences were observed in the Cd contents of wild-type and transgenic plants, and the Co content in the shoots of the transgenic lines was lower than that of the wild type (Figure 7). In the roots, no difference in Pb and Zn contents between the transgenic and wild-type lines was observed. However, in the case of Cd and Co treatments, the Cd and Co contents of OX2 were higher than those of the wild type and OX1. The metal translocation ratios were calculated from these metal contents data. The Pb and Zn translocation ratios in the two transgenic lines were higher than those of the wild type. No significant difference was observed in the Cd translocation ratios between the transgenic and wild-type lines, but the Co translocation ratios of the transgenic lines were significantly lower compared to those of the wild type (Figure 7). These results suggest that overexpression of *CsHMA3* affects the ability of Pb and Zn ion uptake and translocation in *Camelina*.

### Morphological analysis of *CsHMA3*overexpressing transgenic *Camelina*

**Figure 8:**
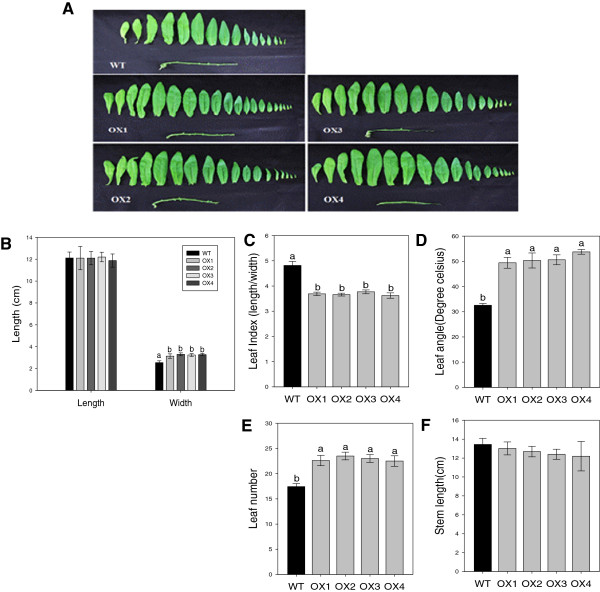
**Different phenotypes of leaves between wild-type and *CsHMA3***-**overexpressing plants at the vegetative stage.** Comparison of shape of leaves from four-week-old plants in hydroponic culture **(A)**. Length of fifth leaf was measured in the leaf-length (longitudinal) and leaf-width (lateral) directions **(B)** and each leaf index was then determined by the ratio of length to width in a leaf blade **(C)**. A value close to 1.0 is indicative of increased leaf roundness. Comparison of leaf angle **(D)**. Each leaf angle was measured in the longitudinal and both lateral-end positions. Leaf count number **(E)**. Leaves were collected from the same plants that were used for investigation of other leaf phenotypes. Measurement of stem length **(F)**. Data represent means ± SD (n = 5). Different lowercase letters in the figure indicate significant difference at *P* < 0.05 by Tukey’s test.

As shown in Additional file 3: Figure S3B, the *CsHMA3* transgenic lines exhibited leaves with a more rounded shape compared to the wild-type leaves. To test whether differences appeared in the leaf shape and phenotype between wild-type and *CsHMA3* transgenic lines at the vegetative (Figure 8) and reproductive stages (Additional file 5: Figure S5), the length, width, angle, number of leaves, and stem length of plants were measured. In both growth stages, remarkable differences in phenotype between the wild-type and *CsHMA3* transgenic lines were observed. Although no difference was observed in the length of leaves between all lines, the width of leaves in the transgenic lines was slightly greater than that of the wild type. In addition, the leaf angle of the *CsHMA3* transgenic lines was markedly greater than that of the wild type. Each leaf angle was measured in the longitudinal and both lateral-end positions. In order to better characterize leaf shape, the leaf index was calculated by ratio of length to width in a leaf blade area. At the vegetative stage, the wild-type and *CsHMA3* transgenic lines had 4.81 ± 0.16 and 3.68 ± 0.03 (mean ± SE of four transgenic lines) leaf index values, respectively. A value close to 1.0 is indicative of increased leaf roundness (Figure 8C). Indeed, the width size (in centimeters) of leaves in the *CsHMA3* transgenic lines was 3.23 ± 0.03 and that of the wild type was 2.5 ± 0.13, but there was no significant difference in the leaf length between the wild-type and *CsHMA3* transgenic lines (Figure 8B). This result indicates that the leaf shape of transgenic lines was rounder than that of the wild type. We also found a similar pattern of leaf index between the wild-type and *CsHMA3* transgenic lines at the reproductive stage (Additional file 5: Figure S5). Furthermore, the leaf numbers of the *CsHMA3* transgenic lines were greater than those of the wild type, but no difference in stem length between the wild-type and *CsHMA3* transgenic lines was observed in both growth stages (Figure 8; Additional file 5: Figure S5).

### Expression pattern of leaf shape related genes between wild-type and *CsHMA3*-overexpressing transgenic *Camelina*

**Figure 9:**
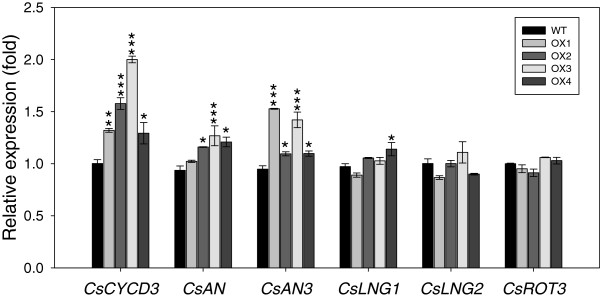
**Gene expression of leaf shape related genes between wild-type and *CsHMA3*-overexpressing transgenic *Camelina. ***Total RNA was extracted from the fifth leaves in wild-type and *CsHMA3* transgenic lines at the vegetative stage. Quantitative real-time PCR was performed to compare the expression levels of the *CsCYCD3, CsAN, CsAN3, CsLNG1, CsLNG2,* and *CsROT3* genes. Expression (fold) was calculated by relative comparison to the expression of the individual genes in the wild type. *CsActin* was used as an internal control for normalization. Values are the averages of three replicates ± SE. Asterisks in the figure indicate significant difference from wild type at *P < 0.05, **P < 0.01, and ***P < 0.001 by Dunnett’s test.

In order to find some clue to the causes of changes in the leaf shape of *CsHMA3*-overexpressing transgenic *Camelina*, the need arose to test whether the expression of leaf shape related genes is affected by the overexpression of *CsHMA3* in *Camelina*. To respond to this question, a targeted expression analysis of several genes that are involved in cell polarity was performed. Representatively, expression patterns of *ANGUSTIFOLIA* (*AN*) and *ANGUSTIFOLIA3* (*AN3*), which are leaf-width direction regulators, and *ROTUNDIFOLIA 3* (*ROT3*), *ROTUNDIFOLIA 4* (*ROT4*), *LONGIFOLIA1* (*LNG1*), and *LONGIFOLIA2* (*LNG2*), which are leaf-length direction regulators, were investigated. The expression of a cell division regulator (*CYCD3*) was also checked as a means to evaluate whether the modification of cell division could be related to the altered leaf shape in *CsHMA3* transgenic lines. Interestingly, transcript levels of cell division regulator and leaf-length direction regulators were induced in *CsHMA3* transgenic lines at the vegetative stage (Figure 9). No significant differences were observed in the expression level of cell proliferation regulator genes between the wild-type and *CsHMA3* transgenic lines*.* These results suggested that the induction of cell division regulator and leaf-length direction regulator transcripts was associated with alterations of leaf -width in *CsHMA3* transgenic lines (Figure 9).

### Analysis of productivity index of *CsHMA3-*overexpressing transgenic *Camelina* treated with heavy metals in a greenhouse

In order to confirm whether soil-grown *CsHMA3*-overexpressing transgenic *Camelina* can also maintain the capacity of heavy metal tolerance for a longer period (90 days), we compared the productivity index between the wild-type and two transgenic lines. The productivity index includes the following: dry weight (g), height (cm), total number of siliques per plant, total seed weight (g), total seed number, and weight (g) per thousand seeds. When plants were grown under control conditions, no significant differences in the six productivity indexes were observed between the wild-type and the two transgenic lines. In the Cd, Pb, and Zn treatment, the dry weight of the two transgenic lines was higher by about 1.3- to 2-fold than that of the wild type, but not in the Co treatment. This is consistent with the heavy metal tolerance phenotype of transgenic lines observed from the vertical plate containing Cd, Pb, and Zn. In all the treatments, the total seed weight and total seed number of the two transgenic lines were significantly greater by about 1.5- to 3-fold compared to the wild type, but no significant difference was observed in weight per thousand seeds between the wild-type and the two transgenic lines (Table 1).

**Table 1:** **Analysis of productivity parameters between wild-type and *CsHMA3***-**overexpressing *Camelina *by metal treatments**

**Treatments**		**Dry weight (g)**	**Height (cm)**	**Total number of siliques**	**Total seed weight (g)**	**Total seed number**	**Weight per 1,000 seeds (g)**
Control	WT	9.12 ± 0.43	78.66 ± 1.54	299.4 ± 22.52	1.72 ± 0.23	2121.8 ± 258.87	0.81 ± 0.01
	OX1	10.08 ± 1.16	74.51 ± 2.04	301 ± 19.03	1.71 ± 0.31	2180.8 ± 310.55	0.77 ± 0.05
	OX2	10.27 ± 1.41	76.75 ± 4.13	307 ± 24.52	1.71 ± 0.37	2074.4 ± 335.51	0.79 ± 0.04
Cd	WT	4.68 ± 0.12	69.37 ± 0.68	156.6 ± 8.23	0.54 ± 0.05	635.8 ± 92.75	0.49 ± 0.01
	OX1	5.85 ± 0.15^**^	72.25 ± 1.65	235.2 ± 12.4^**^	1.09 ± 0.11^*^	1212.4 ± 24.94^***^	0.49 ± 0.01
	OX2	7.02 ± 0.19^***^	67.11 ± 3.01	231.8 ± 12.19^**^	1.37 ± 0.13^**^	1384.2 ± 10.49^***^	0.51 ± 0.02
Pb	WT	4.93 ± 0.38	56.51 ± 3.85	130.2 ± 11.57	0.93 ± 0.06	1015.4 ± 81.28	0.54 ± 0.03
	OX1	7.86 ± 0.42^**^	67.51 ± 2.92^*^	275.6 ± 11.88^***^	1.39 ± 0.09^**^	1550 ± 121.32^**^	0.57 ± 0.01
	OX2	7.48 ± 0.32^**^	69.25 ± 2.05^*^	261.2 ± 10.16^***^	1.35 ± 0.04^**^	1597.6 ± 90.21^**^	0.57 ± 0.01
Zn	WT	4.61 ± 0.32^a^	60.66 ± 3.79	141.4 ± 10.7	0.72 ± 0.09	833 ± 52.39	0.51 ± 0.01
	OX1	9.15 ± 0.61^***^	69.75 ± 1.01	161.2 ± 15.97	1.48 ± 0.07^***^	1602 ± 85.12^***^	0.52 ± 0.01
	OX2	8.81 ± 0.35^***^	71.75 ± 2.81^*^	163 ± 20.23	1.25 ± 0.02^***^	1299.6 ± 25.14^***^	0.51 ± 0.01
Co	WT	4.15 ± 0.55	65.12 ± 3.17	112 ± 10.2	0.65 ± 0.02	644.4 ± 91.46	0.41 ± 0.01
	OX1	3.86 ± 0.36	65.61 ± 3.27	121.8 ± 11.38	1.56 ± 0.06^***^	1543.6 ± 41.03^***^	0.45 ± 0.01
	OX2	4.53 ± 0.25	70.84 ± 1.75	128.6 ± 11.71	1.58 ± 0.09^***^	1588.2 ± 60.97^***^	0.43 ± 0.01

Seeds were germinated and grown on soil conditions containing 60 mg/kg Cd, 6,500 mg/kg Pb, 1,500 mg/kg Zn, 600 mg/kg Co for 90 days. Data represent means ± SD (n = 5). Asterisks indicate significant difference from wild-type subjected to the same treatment and each parameter at *P < 0.05, **P < 0.01 and ***P < 0.001 by Dunnett’s test.

## Discussion

*Camelina* is rich in omega-3 fatty acids, and various stages of research and development are currently being carried out on the use of *Camelina* as a biofuel. The production and utilization of *Camelina* as a biofuel have been limited because of the lack of definite knowledge about resistance to abiotic stresses, despite *Camelina’s* advantages of being able to adapt to a wide range of climates and being able to grow in barren environments that are usually unsuitable for food crops. Even though some research groups have reported that *Camelina* has resistance to diseases, pests, low temperature, and drought stress [6,32-34], to the best of our knowledge, there has been no previous report on heavy metal stress tolerance.

It has been suggested that rapeseed, known as the representative oilseed crop, can possibly be used as a heavy metal extracting plant for phytoremediation [35-37]. To find out whether *Camelina* also has this possibility, we compared the metal tolerance and accumulation between *Camelina* and rapeseed. In a root elongation test under heavy metal stress, *Camelina* plants showed more Cd and Pb tolerance than rapeseed (Figure 1). When plants were exposed to heavy metal stress conditions, a negative effect of heavy metals on membrane integrity and cell permeability occurred and electrolytes were released to the outside of the membrane [38]. Measurement of electrolyte leakage has been used to quantify damages to cell membranes in several abiotic stresses such as drought, chilling and torrid conditions, air pollution, salt stress, acid conditions, and heavy metal exposure [39]. In an electrolyte leakage test, we treated both plants with Cd, Pb, Zn, and Co for five days. *Camelina* leaf showed significantly lower electrolyte leakage level than that of rapeseed leaf under Pb stress, but not under other metal stresses. Furthermore, the Pb, Zn, and Co contents in the *Camelina* shoot were higher than those of rapeseed (Figure 2). The results show that *Camelina* has similar or more tolerance depending on metal species and greater heavy metal translocation ability compared with rapeseed. Therefore, it is suggested that *Camelina* is more suitable than rapeseed to use for phytoremediation when considering heavy metal tolerance and accumulation.

Plants contain metal chelators and transporters that regulate metal homeostasis. The *HMA3* gene is one of several potential candidates involved in mediating metal-hypertolerant and metal-hyperaccumulating traits, and encodes proteins belonging to the P_IB_-type. In addition, high *HMA3* expression levels in shoots of a hyperaccumulator (*T. caerulescens*) have been reported. As a result, metal compartmentation into leaf vacuoles is enhanced, elevating metal translocation [22]. We determined the expression patterns for eight members of P_1B_-ATPases in *Camelina* (root, leaf, stem, flower, and silique) under normal conditions. Among them, the *CsHMA3* gene was expressed in all organs. *CsHMA2* and *CsHMA4*, known as a physiological master switch in metal hyperaccumulation, were detected in the stem and flower (Figure 3A). In addition, *CsHMA3* showed remarkably elevated levels in Pb-treated leaves (Figure 3). We have hypothesized that the high expression of the *CsHMA3* gene might improve heavy metal tolerance and accumulation, especially for Pb. Therefore, we attempted to determine how the CsHMA3 protein can change the ability of heavy metal uptake and translocation in plants. Also, the expression levels of *HMA1* were highly regulated by Pb, Zn, Co, and Cd (Figure 3B). These data also raised the possibility that *CsHMA1* can be used as a potential candidate gene for phyoremediation in future studies.

In this study, we show that *CsHMA3*-overexpressing *Camelina* plants show tolerance against Cd, Pb, and Zn, but not against Co, and that they enhance the accumulation of Pb and Zn in *Camelina* shoots (Figures 5, 6, and 7). We provide several lines of evidence to implicate CsHMA3 in heavy metal tolerance in *Camelina*. First, the *Δycf1* or *Δzrc1* mutant strain yeasts expressing *CsHMA3* were able to complement the growth defect in the presence of 10 μM Pb and 5 mM Zn, but not in the Cd medium (Figure 4). The pattern of growth of transformed yeast cells showed results partly similar to those of the *AtHMA3*-transgenic yeast cell [31]. Functional expression of *AtHMA3* phenotypically complements the Cd/Pb-hypersensitive yeast strain *Δycf1*, but not the Zn-hypersensitive mutant *Δzrc1 *[31]. Although the complementation of yeast growth defect to Cd failed in the expression of *CsHMA3*, our result indicated that CsHMA3 complemented the Pb and Zn sensitivity of yeast mutants. To further support this hypothesis, an additional experiment aiming at the determination of the metal concentration in the yeasts experiments was required. It may be possible to explain that the opposite phenotype of *CsHMA3*-expressing yeast under Cd stress and Pb/Zn stresses was caused by either internal sequestration or metal efflux out of the cell dependent on the metal species. Second, we demonstrate that *CsHMA3*-overexpressing lines grow better than wild-type lines in Cd-, Pb-, or Zn-containing medium or solution (Figure 5A and Figure 6B), and the experimental results can be explained well by differences between root length, fresh weight, and electrolyte leakage between *CsHMA3* transgenic lines and the wild type (Figures 5B, C, and 6A). Third, as shown in Figure 7, it is worth noting that *CsHMA3* transgenic lines mediate efficient translocation of the absorbed Pb and Zn from root to shoot. Although we are not certain about the mechanism of increased translocation in the *CsHMA3* transgenic lines, we propose that CsHMA3 is likely to act as a short-term storage “filter” in the root, unlike AtHMA3 [40,41], thereby less limiting heavy metal translocation to the shoot. This is similar to the proposal for the function of TcHMA3 in *T. caerulescens *[22].

However, CsHMA3 and TcHMA3 seem to have different metal substrate specificities. Overexpression of *CsHMA3* increased the Pb and Zn tolerance and accumulation, but not those of Co. Enhanced tolerance of Cd was also observed in transgenic plants, but no difference in Cd accumulation between wild-type and transgenic lines was observed (Figures 4, 5, 6 and 7). When *TcHMA3* was expressed in either yeast or *Arabidopsis*, it only enhanced tolerance of Cd, but not of Pb and Co. Furthermore, in genome-wide association studies, it was determined that HMA3 drives natural variation occurring in Cd concentration of leaf in *A. thaliana,* and grafting indicated that HMA3 functions in the root to govern shoot Cd translocation [42]. This is consistent with the OsHMA3 limiting translocation of Cd through the xylem into the shoot [43,44]. These results suggest that other HMA homologs in various species have different functions with respect to substrate specificity and toxic metal sequestration. It has been proposed that the metal specificity of P_1B_-ATPases is indicated by conserved amino acids located in transmembrane domain (TMD) 6 at the CPx or SPC sequences, and by signature sequences in TMD7 and TMD8 in P_1B_-ATPases [45]. By analyzing all available P_1B_-ATPase protein sequences, and searching for similarities and conserved amino acid sequences in TMD6, TMD7, and TMD8, signature sequences were identified and the P_1B_-ATPases were divided into four subgroups with distinct metal specificity (1B-1, Cu^+^/Ag^+^; 1B-2, Zn^2+^/Cd^2+^/Pb^2+^/Co^2+^; 1B-3, Cu^2+^/Cu^2+^/Ag^2+^; 1B-4, Cu^2+^) and two subgroups (1B-5 and 1B-6) [17,45]. Although these three specific amino acid regions are identical in HMA3 proteins of *Camelina*, *T. caerulescens*, *A. thaliana* (Ws), and *A. halleri*, the metal-substrate specificity of these plants is not completely matched [22]. Thus, the specific factor related to determining metal-substrate specificity, based on additional structural considerations in the N- and/or C-terminal ends of CsHMA3, requires further study.

Here, we also found that *CsHMA3*-overexpressing *Camelina* plants clearly exhibited a wider and more orbicular leaf blade than the wild type, and leaf numbers in the *CsHMA3* transgenic lines were also greater than in the wild-type lines at both vegetative and reproductive growth stages (Figure 8). This indicated that these leaf phenotypes of *CsHMA3* transgenic lines did not correlate to any difference in growth stages. The shapes and sizes of leaves are crucial factors influencing the life of plants, because leaves are photosynthetic organs. To absorb enough light energy, leaves need to be as wide as possible. However, the leaf area is principally regulated by the availability of water [46]. The formation of leaf shape and size is affected by cell differentiation on the leaf surfaces during the stages of leaf morphogenesis and lateral, two-dimensional expansion of the leaf blade [47]. Generally, these steps are regulated by several leaf shape related genes [47,48]. *Arabidopsis* leaf blades expand in two dimensions, which are described as leaf-length (longitudinal) and leaf-width (lateral) axes. The ratio of the two axes has been used as the standard to establish leaf blade shape [49]. The leaf-width (lateral) and leaf-length (longitudinal) axes of leaf expansion are regulated by the *AN*, and the *ROT* genes, respectively. In addition, *AN3*/*GRF-INTERACTING FACTOR1* (*AtGIF1*) and *SPIKE1* have been known as regulators in the leaf-width direction [50-53]. With respect to the regulation of leaf length, *ROT3*, *ROT4*, *LNG1,* and *LNG2* are involved in the regulation of leaf length polarity growth [54-56]. *Arabidopsis* cold shock domain protein 3 (AtCSP3) may also function to regulate leaf-length direction [49]. The real-time (RT)-PCR data in Figure 9 suggest that a wider leaf shape observed in *CsHMA3* transgenic lines might be caused by inductions in the transcript level of leaf-width direction regulator genes.

The ectopic expression of *CsHMA3* all over the plant body likely modifies the concentration of an essential metal such as Zn in cellular compartments. This modification interacts with the whole plant’s metal-homeostasis regulation and with numerous pathways, leading to changes in the regulation of leaf development and metal translocation to shoots of *CsHMA3* transgenic lines. Thus, we could not assert that these changes directly indicate the physiological function of the CsHMA3. To understand the exact role of CsHMA3, further experiments such as phenotypic analysis of a CsHMA3 knockdown plant and identification of its tissue and cellular location are required.

The aim of our study was to show if *Camelina* could be used for phytoremediation of metal-contaminated soil and in parallel for oil production. However, some crucial problems about the content of heavy metals in the oil obtained from *Camelina* plants grown in heavy metal contaminated areas remained unsolved. Further research to investigate if biofuels containing heavy metals exhaust poisonous gas is required. Also, it is crucial to determine whether overexpression of *HMA3* affects the fatty acids composition, which is responsible for the fuel properties of biodiesel, in plants grown both under control conditions and in the presence of metals. The productivity index analysis of *CsHMA3* transgenic and wild-type lines indicated that *CsHMA3*-overexpressing transgenic plants showed less inhibition of the production of silique by Cd and Pb stress; thus, the total seed number and total seed weight were higher than those of the wild type (Table 1). Even though no difference in total number of siliques was observed between the wild-type and the two transgenic lines under Zn and Co stress, the total seed number and total seed weight in the two transgenic lines were higher than those of the wild type, because there were significantly more seeds per silique. These observations indicate that the *CsHMA3* gene can contribute to increasing the productivity of seeds in the heavy metal contaminated soil.

## Conclusions

In conclusion, our data demonstrated that CsHMA3 affects the Cd, Pb, and Zn tolerance and the ability of heavy metal uptake and root-to-shoot translocation, especially for Pb and Zn in *Camelina*. This provides the possibility for phytoremediation of multiple metal contaminated sites. Also, overexpression of *CsHMA3* resulted in larger leaf size compared with the wild type due to an induction of leaf shape related genes and showed an increasing productivity of seeds in the soil treated with heavy metal. This information will also be useful to improve productivity of bioenergy crops. Moreover, the heterologous overexpression of *HMA3* and *HMA4* in plants is probably a useful strategy to engineer altered metal distribution in tissues for biofortification or phytoremediation purposes [57].

## Materials and methods

### Plant material and growth conditions

Seeds of *Camelina sativa* L. cv. “CAME” and *B. napus* L. cv. “Youngsan” were grown at 22 ± 1°C under long days (16-/8-h day/night light with 200 μmol m^-2^ s^-1^ cycle). To analyze the germination rate and seedling growth under heavy metal stresses, plants were grown vertically on 125 × 125 × 20 mm polystyrene petri dishes containing half-strength Murashige and Skoog medium (Duchefa Biochemie; basal salts mixture) or modified nutrient medium (without phosphate to avoid Pb precipitation) containing 1.25 mM KNO_3_, 1.5 mM Ca(NO_3_)_2_, 0.75 mM MgSO_4_, 75 μM FeEDTA, 50 μM H_3_BO_3,_ 10 μM MnCl, 2 μM ZnSO_4_, 1.5 μM CuSO_4_, and 0.075 μM (NH_4_)₆Mo₇O_24_ containing 0.8% (w/v) phytoagar (Duchefa Biochemie) and 1.5% (w/v) sucrose after sterilization with 75% ethanol for 10 min. CdCl_2_, Pb(NO_3_)_2_, ZnSO_4_, and CoCl_2_ were supplemented into the corresponding media at the concentrations indicated in the Figures 1, 5 and Additional file 4: Figure S4. To reduce the variation due to the precipitation of CdCl_2_, Pb(NO_3_)_2_, ZnSO_4_, and CoCl_2_, the plants were grown in the same plate and their growth was monitored for three weeks. A seed was regarded as germinated when the radicle protruded from the seed coat. Germination and growth assays were carried out on three replicates.

### Measurement of electrolyte leakage

For the measurement of electrolyte leakage, leaves were excised and washed three times with distilled water. After removing the water with filter paper, the leaves were cut into small pieces (about 5 mm) and placed in flasks with 60 mL distilled water. The flasks were shaken at 26˚C for 2 hours [58], and the electrical conductivity of the solution (EC1) was measured using an IQ170 electrical conductivity meter (IQ Scientific Instruments, San Diego, CA, USA). Autoclaved samples (EC2) were measured to determine the maximum percentage of electrolyte leakage. The relative electrical conductivity (REC) was calculated as REC (%) = (EC1/EC2) × 100.

### Expression analysis of *CsHMAs* and leaf shape related genes

To determine the expression of *CsHMAs* and leaf shape related genes by reverse transcription (RT)-PCR, the total RNA was obtained from tissues (flower, stem, leaf, and root) of four- or six-week-old *Camelina* grown on rockwool using the RNAiso Plus Kit (Takara code: D9108A, Shiga, Japan) and the RNeasy Plant Mini Kit (Qiagen, Valenica, CA) according to manufacturer instructions. The quantity of RNA was measured using a spectrophotometer (Nanovue, Daejeon, Korea), and its quality was checked using agarose gel electrophoresis. RNA was treated with RNase-free DNase-I (Ambion, Austin, TX) for 45 min followed by DNase-I removal as specified by the manufacturer. Two micrograms of DNA-free RNA were then reverse transcribed using the First-Strand Synthesis System (Invitrogen) for RT-PCR according to the manufacturer’s specifications. cDNA concentrations were normalized with *CsActin* or *beta-tubulin*. To amplify *CsHMA1* to about eight cDNAs, PCRs were performed in a final volume of 20 μL using cDNA as templates in a PTC-100 Peltier Thermal Cycler (MJ Research, Waltham, MA) using specific primers (Additional file 6: Table S1). Cycling conditions were 5 min at 95°C, followed by 25 or 30 cycles of 15 s at 95°C, 30 s at 55.3 or 57.3°C, and 30 s at 72°C. At the end of the cycling, the samples were incubated at 4°C. For comparative quantification of leaf shape related genes, real-time quantitative PCR (qRT-PCR) was performed in a Bio-Rad CFX96 Real-Time PCR system (Bio-Rad Laboratories Inc., Hercules, CA, USA). The 20-μL PCR reaction mixture contained 4 μL of diluted cDNA, 10 μL of SYBR Premix (Takara), 2 μL of a mix of the primer pair, and 4 μL of water. The real-time PCR condition was followed by a three-step protocol with melting curve. The data were analyzed using the Bio-Rad CFX manager (version 1.0) after normalization to *CsActin*. Triplicate PCR and three biological replicates were analyzed. The primers used for this experiment are listed in Additional file 6: Table S1.

### Vector construction and *Camelina* transformation

☐Total RNA was isolated from two-week-old seedlings using the RNAiso Plus Kit (Takara code: D9108A, Shiga, Japan). RNA was treated with RNase-free DNase-I (Ambion, Austin, TX) for 45 min followed by DNase-I removal as specified by the manufacturer, and the cDNA was synthesized using the SMARTerTM RACE cDNA Amplication Kit (Clontech, Mountain View, CA). The full-length coding sequence of CsHMA3 was obtained using 3’ and 5’ rapid amplification of cDNA ends (RACE Technique, Clontech) according to the protocol of the SMART™ RACE kit (Clontech). Full-length cDNA was cloned using 5′-AAAAAGCAGGCTATGGTGGAAGGTGAAGAGACAAAG-3′ and 5′-AGAAAGCTGGGTTCACTTTTGTTGATCCTCCTTAGGGCC-3′ primers. Two recombination sites, attB1 and attB2 at the 5’-ends, were added to the forward and reverse primer, respectively. The pair of PCR primers was not specific for only *CsHMA3a* full sequence amplification. The PCR products were purified and cloned into the pGEM-T Vector System (Promega, Madison, WI). Seven clones were obtained and sequenced. Among them, sequences of four clones were consistent with each other. This sequence has been deposited in GenBank [GenBank:JX402100] (*CsHMA3a*). Subsequently, this gene was cloned in pDONR207 using the Gateway Cloning System (Invitrogen). The binary vector pCB302-3 [59] contained the promoter and terminator of the CaMV (35 s promoter) to overexpress a *CsHMA3* cDNA in *Camelina* and was modified to be compatible with the Gateway system as described by the manufacturer. Clones from which the expected sequence was confirmed were transferred to the pCB302-3 vectors. The transformation of *Camelina* was conducted via vacuum infiltration while immersing flowers for 5 min in the *Agrobacterium*-containing solutions [60]. *Camelina* plants at the early flowering stages were used for transformation. To get a high ratio of transformation, plants were used three times for transformation at four-day intervals. Selection was performed in *E. coli* and *A. tumefaciens* strains with 60 μg/mL kanamycin and in plants with 50 mg/mL DL-phosphinotricin (PPT) (Duchefa, Harlem, The Netherlands). The frequency of transformation was about 0.5%.

### Yeast transformation and Zn/Cd tolerance analysis

The wild-type *S. cerevisiae* strain BY4741 (EUROSCARF accession number Y0000; *MATa*; *his3Δ1*; *leu2Δ0*; *met15Δ0*; *ura3Δ0*), a BY4741-derived Cd/Pb-sensitive strain with *Δycf1* (Y04069), and a Zn-sensitive strain with *Δzrc1* (Y00829) were used for complementation analysis of *CsHMA3*. The pYES2*-CsHMA3* expression constructs were generated by inserting *CsHMA3* expression cassettes into the *BamH*I/*Xho*I sites of pYES2 (driven by the GAL1 promoter). The construct was then introduced into the wild-type yeast strain BY4741, the Cd/Pb-sensitive *Δycf1* strain, and the Zn-sensitive *Δzrc1* yeast strain using the lithium acetate method [61]. Yeast cells transformed with empty pYES2 were used as a control. Transformants were selected on 2% glucose, 0.67% Yeast Nitrogen Base (without amino acids, Difco), 0.16% Yeast Synthetic Drop-Out Medium Supplement Without Uracil (Sigma-Aldrich), and 1.5% bacto agar. For the metal tolerance assays, yeast cells grown in the log phase were diluted to an OD_600_ of 1, 10^-1^, 10^-2^, 10^-3^, and 10^-4^, and 5 μL of each dilution were spotted onto LPM medium containing galactose without or with added CdSO_4_, PbNO_3_, and ZnSO_4_. The plates were incubated at 30°C for four days before photography. The yeast cells were grown in the presence of 25 μM Cd, 10 μM Pb, and 5 mM Zn.

### Measurements of metal contents

Plants were grown on rockwool in a 4-L container volume (six or nine plants per one container) containing aerated nutrient solution for about three to four weeks. The nutrient solution contained 1.25 mM KNO_3_, 1.5 mM Ca(NO_3_)_2_, 0.75 mM MgSO_4_, 0.5 mM KH_2_PO_4_, 75 μM FeEDTA, 50 μM H_3_BO_3,_ 10 μM MnCl, 2 μM ZnSO_4_, 1.5 μM CuSO_4_, and 0.075 μM (NH_4_)₆Mo₇O_24_. The solutions were supplemented every day and changed once a week or to avoid the excessive depletion of any particular ion. For heavy metal treatments, plants were transferred to a solution with concentrations of CdCl_2_, Pb(NO_3_)_2_, ZnSO_4_, or CoCl_2_, as indicated in the Figures 2 and 7. In the case of Pb treatment, plants were transferred to phosphate-free nutrient solution at least 12 hours prior to treatment. After growing for seven days, plant tissues (root and shoot) were harvested, rinsed in ice-cold 1 mM tartarate solution, and blot-dried at 60°C for three days. Each dried sample was weighed and digested with 11 N HNO_3_ at 200°C using a microwave digestion system (MDS Ethos1, Milestone, Italy). Digested samples were diluted with 0.5 N HNO_3_ and then analyzed using inductively coupled plasma mass spectroscopy with an IRIS-AP apparatus (Thermo Scientific, Pittsburgh, PA). The rate of metal translocation was estimated as the percentage of metal content in the shoot compared with that in the whole plant [44].

### Statistical analysis

Statistical analyses for biological data were carried out using the SPSS statistical software (SPSS, Chicago, IL, USA). Statistical differences between measurements (n = 3, 5, or 30) for different results were analyzed following Student’s *t-*test, Dunnett’s test, and Tukey’s test. Values of *P* < 0.05, *P* < 0.01, or *P* < 0.001 were considered statistically significant.

## Abbreviations

HMA: heavy metal ATPase; RACE: rapid amplification of cDNA ends; OX: overexpression.

## Competing interests

The authors declare that they have no competing interests.

## Authors’ contributions

WP planned the study, carried out the experiment, and drafted the manuscript. SJA planned the study and revised the manuscript. YF performed the investigation of productive parameters in the wild-type and *CsHMA3* transgenic lines. All authors read and approved the final manuscript.

## Supplementary Material

Additional file 1: Figure S1 Effect of metal treatments on the electrolyte leakage of the leaves of *Camelina* and rapeseed. Four-week-old plants were grown in nutrient solution hydroponically and treated with 50 μM Cd, 500 μM Pb, 500 μM Zn, and 100 μM Co for five days. In the case of Pb treatment, plants were transferred to phosphate-free nutrient solution at least 12 hours prior to treatment. Data represent means ± SD (n = 6). Asterisks in the figure indicate significant difference between *Camelina* and rapeseed subjected to the same treatment at *P < 0.05 by Student’s *t-*test. Click here for file

Additional file 2: Figure S2 Comparison of the amino acid sequences of three CsHMA3 copies according to Clustal W multiple alignment (version 1.83). The gray box shows metal binding domain (MBD), and lines over sequence indicate putative transmembrane domain (TMD), P-type ATPase signature (PAS), and HMA signature (HMAS). The red arrow indicates the position of the stop codon observed in CsHMA3c. Click here for file

Additional file 3: Figure S3 Confirmation of *CsHMA3* overexpression lines. *CsHMA3* transcript levels of five *CsHMA3* transgenic T_2_ plants (H3-1, 3-2, 3-3, 3-5, and 3-7) and wild type were determined by RT-PCR **(A)**. Different phenotypes of leaves between wild-type and *CsHMA3*-overexpressing plants **(B)**. Click here for file

Additional file 4: Figure S4 Effect of Co stress on root growth in *Camelina* wild-type and transgenic lines. Seeds were germinated and grown on agar plates containing different concentrations of heavy metals for seven days. Photographs taken of *Camelina* wild-type and two *CsHMA3*-overexpressing lines grown on agar plates containing different concentrations of Co for one week **(A)**. Root growth rate **(B)** of the plants was measured at the seventh day after heavy metal treatment. Data represent means ± SD (n = 30). Click here for file

Additional file 5: Figure S5 Different phenotypes of leaves between wild-type and *CsHMA3*-overexpressing plants at the reproductive stage. Comparison of leaf shape of tenth leaf from plants 42 days after germination (DAG) in hydroponic culture **(A)**. Leaf length was measured in the leaf-length (longitudinal) and leaf-width (lateral) directions. Comparison of leaf index **(B)**. Each leaf index was determined by the ratio of length to width in a leaf blade. A value close to 1.0 is indicative of increased leaf roundness. Comparison of leaf angle **(C)**. Each leaf angle was measured in the longitudinal and both lateralend positions. Leaf count number **(D)**. Leaves were collected from the same plants that were used for investigation of other leaf phenotypes. Measurement of stem length **(E)** (n = 5). Different lowercase letters in the figure indicate significant difference at *P* < 0.05 by Tukey’s test. Click here for file

Additional file 6: Table S1 Primer sequence list used in this study. Click here for file
