# Supplementary material for: Alteration of leaf shape, improved metal tolerance, and productivity of seed by overexpression of CsHMA3 in Camelina sativa
Source: Biotechnol Biofuels. 2014 Jun 22;7:96. doi: 10.1186/1754-6834-7-96 (PMC4094532; doi:10.1186/1754-6834-7-96)
Supplement: Additional file 1: Figure S1 — Effect of metal treatments on the electrolyte leakage of the leaves of Camelina and rapeseed. Four-week-old plants were grown in nutrient solution hydroponically and treated with 50 μM Cd, 500 μM Pb, 500 μM Zn, and 100 μM Co for five days. In the case of Pb treatment, plants were transferred to phosphate-free nutrient solution at least 12 hours prior to treatment. Data represent means ± SD (n = 6). Asterisks in the figure indicate significant difference between Camelina and rapeseed subjected to the same treatment at *P < 0.05 by Student’s t-test. [file 1754-6834-7-96-S1.docx]

**Additional file 1 – Figure S1. Effect of metals treatments on the electrolyte leakage of the leaves of *Camelina* and rapeseed.** Four-week-old plants were grown in nutrient solution hydroponically and treated with 50 µM Cd, 500 µM Pb, 500 µM Zn, and 100 µM Co for 5 days. In case of Pb treatment, pants were transferred to phosphate-free nutrient solution at least 12 hours prior to treatment. Data represent means ± SD (n=6). Asterisks in the figure indicate significant difference between *Camelina* and rapeseed subjected to the same treatment at *P < 0.05 by Student’s *t* test.
