# Supplementary material for: Alteration of leaf shape, improved metal tolerance, and productivity of seed by overexpression of CsHMA3 in Camelina sativa
Source: Biotechnol Biofuels. 2014 Jun 22;7:96. doi: 10.1186/1754-6834-7-96 (PMC4094532; doi:10.1186/1754-6834-7-96)
Supplement: Additional file 2: Figure S2 — Comparison of the amino acid sequences of three CsHMA3 copies according to Clustal W multiple alignment (version 1.83). The gray box shows metal binding domain (MBD), and lines over sequence indicate putative transmembrane domain (TMD), P-type ATPase signature (PAS), and HMA signature (HMAS). The red arrow indicates the position of the stop codon observed in CsHMA3c. [file 1754-6834-7-96-S2.docx]

**MBD**

**TMD1**

**TMD2**

**STOP**

**TMD3**

**TMD4**

**TMD5**

**TMD6**

**PAS**

**HMAS**

**TMD8**

**TMD7**

**Additional file 2 – Figure S2. Comparison of the amino acid sequences of three CsHMA3 copies according to CLUSTAL W multiple alignment (version 1.83).** Grey box shows metal binding domain (MBD), and lines over sequence indicate putative transmembrane domain (TMD), P-type ATPase signature (PAS), and HMA signature (HMAS). The red arrow indicates the position of the stop codon observed in CsHMA3c.
