# Supplementary material for: Alteration of leaf shape, improved metal tolerance, and productivity of seed by overexpression of CsHMA3 in Camelina sativa
Source: Biotechnol Biofuels. 2014 Jun 22;7:96. doi: 10.1186/1754-6834-7-96 (PMC4094532; doi:10.1186/1754-6834-7-96)
Supplement: Additional file 3: Figure S3 — Confirmation of CsHMA3 overexpression lines. CsHMA3 transcript levels of five CsHMA3 transgenic T2 plants (H3-1, 3-2, 3-3, 3-5, and 3-7) and wild type were determined by RT-PCR (A). Different phenotypes of leaves between wild-type and CsHMA3-overexpressing plants (B). [file 1754-6834-7-96-S3.docx]

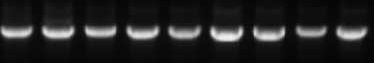


*CsHMA3*

*CsTubulin*

3-1 3-2 3-3 3-4 3-5 3-6 3-7 3-8 WT


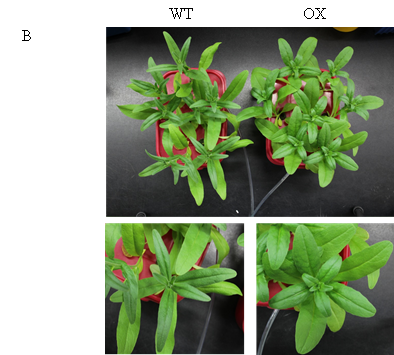


**A**

**B**


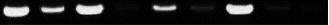


**Additional file 3 - Figure S3. Confirmation of *CsHMA3* overexpression lines.** *CsHMA3* transcript levels of five *CsHMA3* transgenic T_2_ plants (3-1, 3-2, 3-3, 3-5, and 3-7) and wild-type were determined by RT-PCR (A). Different phenotypes of leaves between wild-type and *CsHMA3* overexpressing plants (B).
