# Supplementary material for: Alteration of leaf shape, improved metal tolerance, and productivity of seed by overexpression of CsHMA3 in Camelina sativa
Source: Biotechnol Biofuels. 2014 Jun 22;7:96. doi: 10.1186/1754-6834-7-96 (PMC4094532; doi:10.1186/1754-6834-7-96)
Supplement: Additional file 4: Figure S4 — Effect of Co stress on root growth in Camelina wild-type and transgenic lines. Seeds were germinated and grown on agar plates containing different concentrations of heavy metals for seven days. Photographs taken of Camelina wild-type and two CsHMA3-overexpressing lines grown on agar plates containing different concentrations of Co for one week (A). Root growth rate (B) of the plants was measured at the seventh day after heavy metal treatment. Data represent means ± SD (n = 30). [file 1754-6834-7-96-S4.docx]

WT OX1 OX2

WT OX1 OX2


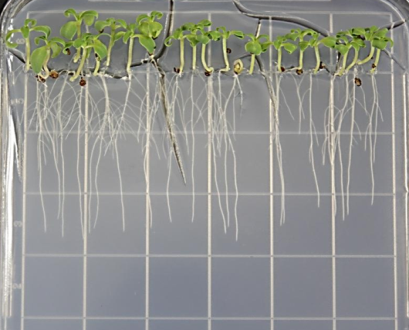


WT OX1 OX2


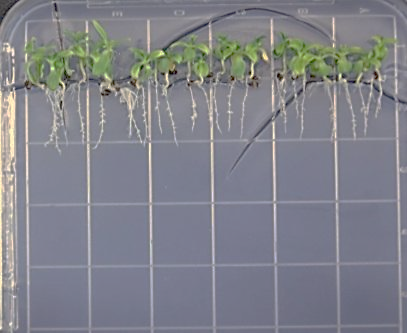

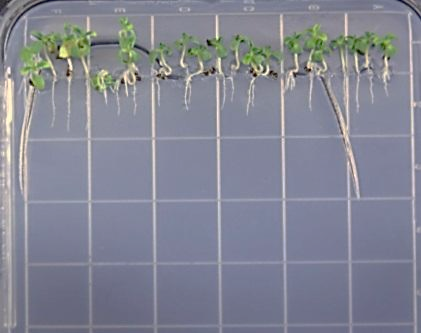


**Additional file 4 - Figure S4.** **Effect of Co stress on root growth in *Camelina* wild-types and transgenic lines.** Seeds were germinated and grown on agar plates containing different concentrations of heavy metals for 7 days. Photographs of *Camelina* wild-type and two *CsHMA3* overexpressing lines were grown on agar plates containing different concentrations of Co for 1 week (A). Root growth rate (B) of the plants was measured at the 7^th^ day after heavy metal treatment. Data represent means ±SD (n=30).
