# Supplementary material for: Alteration of leaf shape, improved metal tolerance, and productivity of seed by overexpression of CsHMA3 in Camelina sativa
Source: Biotechnol Biofuels. 2014 Jun 22;7:96. doi: 10.1186/1754-6834-7-96 (PMC4094532; doi:10.1186/1754-6834-7-96)
Supplement: Additional file 5: Figure S5 — Different phenotypes of leaves between wild-type and CsHMA3-overexpressing plants at the reproductive stage. Comparison of leaf shape of tenth leaf from plants 42 days after germination (DAG) in hydroponic culture (A). Leaf length was measured in the leaf-length (longitudinal) and leaf-width (lateral) directions. Comparison of leaf index (B). Each leaf index was determined by the ratio of length to width in a leaf blade. A value close to 1.0 is indicative of increased leaf roundness. Comparison of leaf angle (C). Each leaf angle was measured in the longitudinal and both lateralend positions. Leaf count number (D). Leaves were collected from the same plants that were used for investigation of other leaf phenotypes. Measurement of stem length (E) (n = 5). Different lowercase letters in the figure indicate significant difference at P < 0.05 by Tukey’s test. [file 1754-6834-7-96-S5.docx]

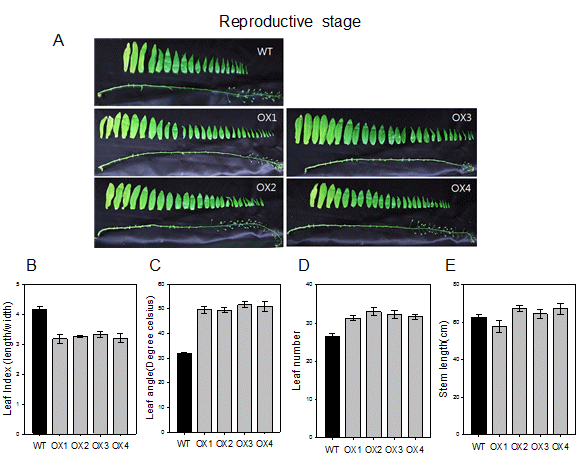


**A**

**B**

**C**

**D**

**E**

**Additional file 5 - Figure S5. Different phenotypes of leaves between wild-type and *CsHMA3* overexpressing plants at the reproductive stage.** Comparison of leaf shape of 10^th^ leaves from 42 DAG plant in hydroponic culture (A). Leaf length was measured in the leaf-length (longitudinal) and leaf-width (lateral) directions. Comparison of leaf index (B). Each leaf index was determined by ratio of length to width in a leaf blade. A value close to 1.0 is indicative of increased leaf roundness. Comparison of leaf angle (C). Each leaf angle was measured in the longitudinal- and both lateral- end positions. Leaf count number (D). Leaves were collected from the same plants that were used for investigation of other leaf phenotypes. Measurement of stem length (E). Data represent means ±SD (n=5). Different lowercase letters in the figure indicate significant difference at P < 0.05 by Tukey’s test.
