# Supplementary material for: Alteration of leaf shape, improved metal tolerance, and productivity of seed by overexpression of CsHMA3 in Camelina sativa
Source: Biotechnol Biofuels. 2014 Jun 22;7:96. doi: 10.1186/1754-6834-7-96 (PMC4094532; doi:10.1186/1754-6834-7-96)
Supplement: Additional file 6: Table S1 — Primer sequence list used in this study. [file 1754-6834-7-96-S6.docx]

| Gene | Accession  Number  [Genebank] | Primer | |
| --- | --- | --- | --- |
| *CsHMA1* | KJ708746 | Forward | 5'-AAGACTTTGTTCATGCCGC–3' |
|  |  | Reverse | 5'-CCTCTGGCTTTAGGTTGCAG–3' |
| *CsHMA2* | KJ708747 | Forward | 5'-CTCAAGTCCTGCTGATCCCG–3' |
|  |  | Reverse | 5'-GTGTGGTGGTGATGGTGACT–3' |
| *CsHMA3* | JX402100 | Forward | 5'-TCGTGAAGGCATTGAATCAA–3' |
|  |  | Reverse | 5'-CAAGGATGGGGAAAACTCCG–3' |
| *CsHMA4* | KJ708748 | Forward | 5'-AGCCTGTTACCTTGGTGGTT–3' |
|  |  | Reverse | 5'-TGCATAACTCCTGCAACAGT–3' |
| *CsHMA5* | KJ708749 | Forward | 5'-GAGATTCAGCGAGCGTTACC–3' |
|  |  | Reverse | 5'-CAACGCATCGATGACAGCTT–3' |
| *CsHMA6* | KJ708750 | Forward | 5'-GGCGGGAGTGTTGCTACCAT–3' |
|  |  | Reverse | 5'-TCTTTTGGTTCCCATTTGAC–3' |
| *CsHMA7* | KJ708751 | Forward | 5'-CGGAGTTACCGGTATGACTT–3' |
|  |  | Reverse | 5'-CTCAGCTTCAAATCCAGCAT–3' |
| *CsHMA8* | KJ708752 | Forward | 5'-TGCACAAGGTAATGCAGCTC–3' |
|  |  | Reverse | 5'-CCAAAGCATCTCCATCAGGT–3' |
| *CsCYCD3* | KJ708753 | Forward | 5'-TCGTTGAACAGTCCAAGCTG–3' |
|  |  | Reverse | 5'-TGCAAAATCGTCTTCTTCGT–3' |
| *CsAN* | KJ708754 | Forward | 5'-CAGTGCTGAGGTCGTTCGTA–3' |
|  |  | Reverse | 5'-CCTCCGCTTTTTGCATGTAT–3' |
| *CsAN3* | KJ708755 | Forward | 5'-CTGCTGGTGGTGGGATGATT–3' |
|  |  | Reverse | 5'-ATTCCAAGCTGGCTATGGTG–3' |
| *CsLNG1* | KJ708756 | Forward | 5'-GTCATTCCGAGGAAGCAGAG–3' |
|  |  | Reverse | 5'-GGCTGACACTAGGCTTCTGG–3' |
| *CsLNG2* | KJ708757 | Forward | 5'-CTCCTCGAGCTTCTCGTCTG–3' |
|  |  | Reverse | 5'-GCTGAGTGAAGGCTGCTTCT–3' |
| *CsROT3* | KJ708758 | Forward | 5'-CGAAGCTCGAAATAGCCATC–3' |
|  |  | Reverse | 5'-CTGAGATGGGAACCATAGCC–3' |
| *CsActin* | KJ708759 | Forward | 5'-GAAGAACTACGAATTACCCG–3' |
|  |  | Reverse | 5'-CGATCCAGACACTGTACTTC–3' |
| *CsTubulin* | KJ708760 | Forward | 5'-CACCTCAAGAGGTTCTCAGC–3' |
|  |  | Reverse | 5'-GTTCAGCATCTGCTCGTCAA–3' |
| *CsHMA3 Full* | | Forward | 5'-AAAAAGCAGGCTATGGTGGAAGGTGAAGAGACAAAG–3' |
|  |  | Reverse | 5'-AGAAAGCTGGGTTCACTTTTGTTGATCCTCCTTAGGGCC–3' |
| 35S promoter | | Forward | 5'-ACGTAAGGGATGACGCACAATC–3' |

**Additional file 6 – Table S1. Primer sequence list used in this study**
